# Contrast‐enhanced harmonic endoscopic ultrasound (CH‐EUS) MASTER: A novel deep learning‐based system in pancreatic mass diagnosis

**DOI:** 10.1002/cam4.5578

**Published:** 2023-01-06

**Authors:** Anliu Tang, Li Tian, Kui Gao, Rui Liu, Shan Hu, Jinzhu Liu, Jiahao Xu, Tian Fu, Zinan Zhang, Wujun Wang, Long Zeng, Weiming Qu, Yong Dai, Ruirui Hou, Shoujiang Tang, Xiaoyan Wang

**Affiliations:** ^1^ Department of Gastroenterology The Third Xiangya Hospital of Central South University Changsha China; ^2^ Hunan Key Laboratory of Nonresolving Inflammation and Cancer Central South University Changsha China; ^3^ Wuhan EndoAngel Medical Technology Co., Ltd. Wuhan China; ^4^ Department of Gastroenterology Zhuzhou Central Hospital Zhuzhou China; ^5^ Department of Gastroenterology The First Affiliated Hospital of University of South China Hengyang China; ^6^ Department of Gastroenterology General Hospital of Ningxia Medical University Ningxia China; ^7^ Division of Digestive Diseases, Department of Medicine University of Mississippi Medical Center Jackson United States

**Keywords:** artificial intelligence, endoscopic ultrasound‐guided fine‐needle aspiration, harmonic contrast‐enhanced endoscopic ultrasound, pancreatic cancer, pancreatitis

## Abstract

**Background and Aims:**

Distinguishing pancreatic cancer from nonneoplastic masses is critical and remains a clinical challenge. The study aims to construct a deep learning‐based artificial intelligence system to facilitate pancreatic mass diagnosis, and to guide EUS‐guided fine‐needle aspiration (EUS‐FNA) in real time.

**Methods:**

This is a prospective study. The CH‐EUS MASTER system is composed of Model 1 (real‐time capture and segmentation) and Model 2 (benign and malignant identification). It was developed using deep convolutional neural networks and Random Forest algorithm. Patients with pancreatic masses undergoing CH‐EUS examinations followed by EUS‐FNA were recruited. All patients underwent CH‐EUS and were diagnosed both by endoscopists and CH‐EUS MASTER. After diagnosis, they were randomly assigned to undergo EUS‐FNA with or without CH‐EUS MASTER guidance.

**Results:**

Compared with manual labeling by experts, the average overlap rate of Model 1 was 0.708. In the independent CH‐EUS video testing set, Model 2 generated an accuracy of 88.9% in identifying malignant tumors. In clinical trial, the accuracy, sensitivity, and specificity for diagnosing pancreatic masses by CH‐EUS MASTER were significantly better than that of endoscopists. The accuracy, sensitivity, specificity, positive predictive value, and negative predictive value were respectively 93.8%, 90.9%, 100%, 100%, and 83.3% by CH‐EUS MASTER guided EUS‐FNA, and were not significantly different compared to the control group. CH‐EUS MASTER‐guided EUS‐FNA significantly improved the first‐pass diagnostic yield.

**Conclusion:**

CH‐EUS MASTER is a promising artificial intelligence system diagnosing malignant and benign pancreatic masses and may guide FNA in real time.

Trial registration number: NCT04607720.

## Graphical abstract

This study was to construct a deep‐learning based system, CH‐EUS MASTER, for facilitating diagnosing pancreatic masses in contrast‐enhanced harmonic endoscopic ultrasonography (CH‐EUS), and for guiding EUS‐guided fine‐needle aspiration (EUS‐FNA) in real‐time, to improve the ability of distinguishing between malignant and benign pancreatic masses. The CH‐EUS MASTER system is composed of Model 1 (real‐time capture and segmentation) and Model 2 (benign and malignant identification). It was developed using deep convolutional neural networks and RandomForest algorithm. In clinical trial, the accuracy, sensitivity and specificity for diagnosing pancreatic masses by CH‐EUS MASTER were significantly better than that of endoscopists. CH‐EUS MASTER guided EUS‐FNA significantly improved the first pass diagnostic yield.

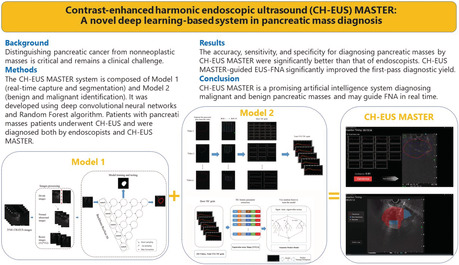

## 1 INTRODUCTION

Distinguishing pancreatic cancer from nonneoplastic masses is critical and remains a clinical challenge. As pancreatic cancer and benign masses, primarily mass‐forming chronic pancreatitis, appear similar in imaging examinations. For patients with pancreatic cancer who do not receive timely diagnosis or present with distant metastases, the median survival is 8–12 months and 3–6 months, respectively.^1^ On the other hand, between 5% and 35% of mass‐forming chronic pancreatitis is misdiagnosed as PC,^2^ causing significant psychological and physical trauma to patients who are needlessly subjected to pancreatoduodenectomy.^3^ Endoscopic ultrasonography (EUS) is one of the most sensitive imaging examinations for the diagnosis of pancreatic masses, but its diagnostic specificity remains limited because most pancreatic cancers and some inflammatory masses appear as low‐echo crumb images.^4^ Cytological examination of EUS‐guided fine‐needle aspiration (EUS‐FNA) is the gold standard of diagnosis. A meta‐analysis including 33 studies showed that the pooled sensitivity for malignant cytology was 85%, and pooled specificity was 98%.^5^ However, the positive puncture site is sometimes difficult to locate and diagnostic accuracy is dependent on endoscopist's experience. As a result, number of patients cannot be diagnosed in a timely or accurate manner.

Contrast‐enhanced harmonic endoscopy ultrasonography (CH‐EUS) is a promising technology to distinguish between malignant and benign pancreatic masses.^6^ CH‐EUS uses a contrast agent combining with tissue harmonic imaging technology to differentiate blood flow characteristics within the benign and malignant pancreatic masses. The pooled sensitivity, specificity, and diagnostic odds ratios of CE‐EUS for the differential diagnosis of PC are 91%, 86%, and 69.50, respectively.^4^ A time–intensity curve (TIC) can be generated based on the temporal change in echo enhancement intensity. Evidence shows that CH‐EUS using TIC analysis is highly effective in differentiating various pancreatic pathologies.^7^ The TIC for pancreatic cancer remains low and flat because the fibrotic and desmoplastic nature of the tumor limits blood supply resulting in an area under the curve that is reduced compared to normal tissues.^8^ Unfortunately, there is still no uniform TIC standard because varying parameter values have been reported in different studies. When CH‐EUS is combined with EUS‐FNA, the sensitivity of EUS‐FNA increases, because CH‐EUS may help to avoid the puncture of necrotic areas (no enhancement) and inflammatory areas (iso‐ or hyperenhancement), which can be present in the center or around the malignant mass, respectively.^8^, ^9^ However, the naked eye is not reliable in identifying and differentiating black and white ultrasonic images, and the endoscopist operating experience may affect the judgment of the results. Moreover, CH‐EUS has a high learning curve and requires technical mastery and strong knowledge of abdominal organ anatomy, limiting its broader application.^10^ For these reasons, there is an urgent need for new technologies that can objectively identify and classify CH‐EUS images to assist diagnosis.

In recent years, computer‐aided diagnosis (CAD) systems have become increasingly sophisticated tools in medical imaging.^11^, ^12^ Image processing and artificial intelligence (AI) have been applied in various areas of medical practice with promising results.^13^ Deep learning is a process of constructing a neural network model analogous to analytical learning in the human brain. Among various kinds of neural networks, deep convolutional neural networks (DCNN) perform well in the recognition and segmentation of features in images.^14^ For example, a DCNN system used in gastroscopy can diagnose gastric cancer quickly using numerous images,^15^ which may relieve endoscopist's clinical burden. As for EUS, a retrospective study investigated whether AI via deep learning algorithms using EUS images of intraductal papillary mucinous neoplasms (IPMNs) could predict malignancy and proved clinicians could make more accurate and faster diagnoses using this tool.^16^ Previous researchers have used artificial neural networks in CH‐EUS imaging systems and obtained appropriate parameters from the TIC for the identification of benign and malignant pancreatic masses.^17^ However, the analysis was performed using commercial software separate from the main engine of the endoscopic ultrasound system, and was not able to track the region of interest (ROI) for real‐time analysis or guide EUS‐FNA during the procedure. Currently, there are few reports about AI recognition and localization of lesions under EUS, and more work in this area is needed.

In this study, we built an auxiliary diagnosis system (CH‐EUS MASTER) onto the main engine of the EUS system in order to identify and track the pancreatic masses dynamically in real time via describing TIC characteristics of each area of the pancreas, identifying points of interest and guiding EUS‐FNA. A single‐center randomized controlled trial was conducted to evaluate whether CH‐EUS MASTER could improve the accuracy of diagnosis for benign and malignant pancreatic masses and improve the diagnostic yield of EUS‐FNA.

## 2 MATERIALS AND METHODS

The CH‐EUS MASTER based on DCNN and Random Forest was designed to achieve three functions: (1) Capturing and segmenting the CH‐EUS pancreatic mass image in real time; (2) Assisting in identifying the benign and malignant of CH‐EUS pancreatic masses according to TIC characteristics; and (3) Guiding targeted EUS‐FNA. All authors had access to the study data and reviewed and approved the final manuscript.

### 2.1 Data sets and preprocessing

A retrospective multicenter collection of 4342 CH‐EUS images was used for training and testing of the Model 1, and 296 stable CH‐EUS videos were used for training and testing of the Model 2 taken from 296 patients with typical pancreatic masses undergoing CH‐EUS examination between January 2018 and December 2019. It was divided into a training set (including tuning set) and independent testing set at a ratio of 8:2. It was confirmed by surgical pathology that 760 cases of pancreatic cancer had 3546 images, and 190 cases of benign pancreatic masses had 886 images in the Model 1 data set. At the same time, there were 167 cancer videos and 128 pancreatitis videos in the Model 2 data set.

One expert in EUS, who has performed >500 EUS‐FNA/FNB, used VGG Image Annotator (VIA) software to enclose the lesion area, and marked benign and malignant lesions according to the pathology and follow‐up, which were provided for use in the machine learning model.

### 2.2 Training and testing of models

#### 2.2.1 Training the model for pancreatic mass image segmentation (Model 1)

**FIGURE 1:**
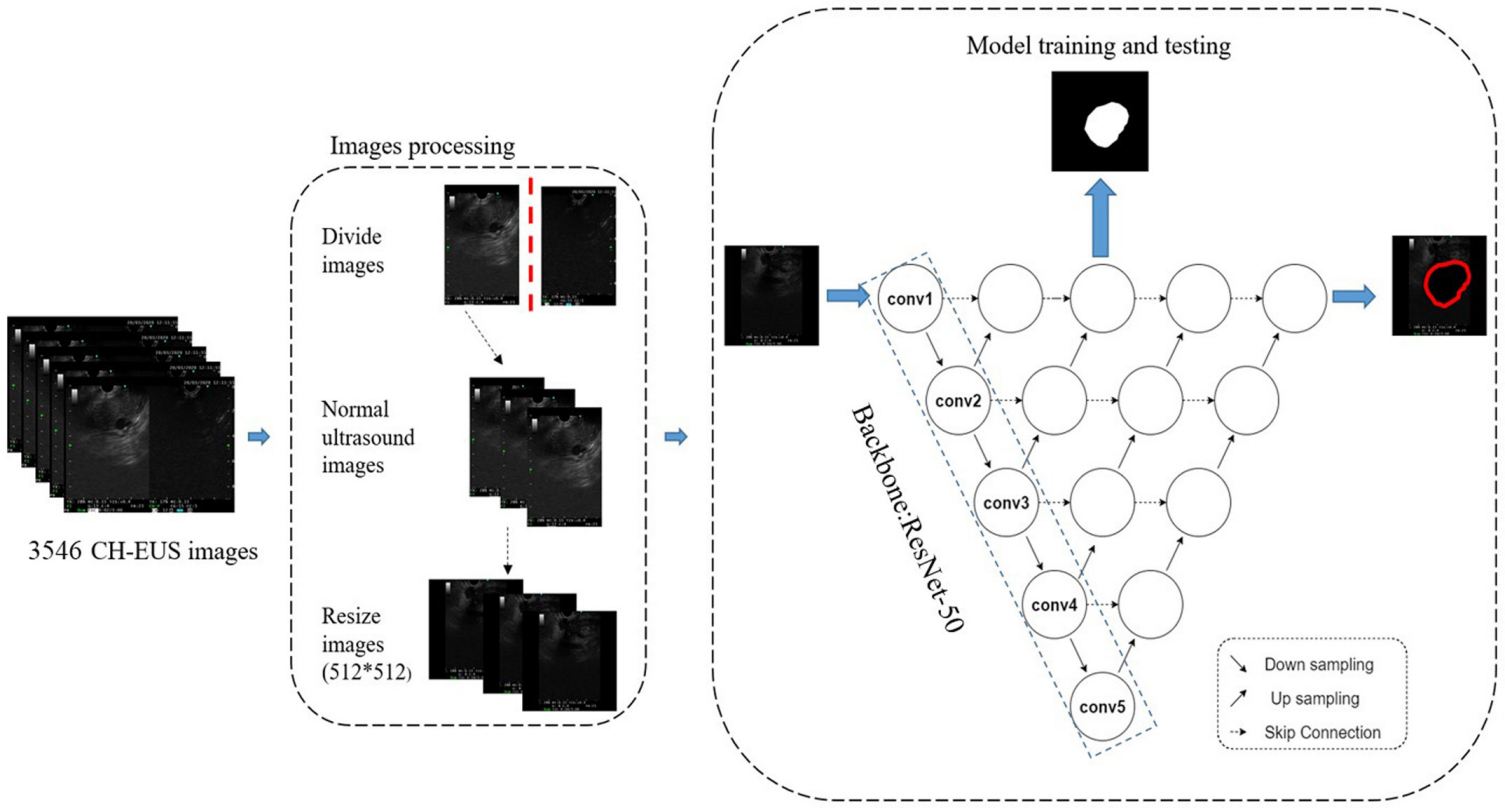
Schematic diagram of training and testing process of the model for pancreatic mass image segmentation (Model 1).

Model 1 was built on the top of UNet++, a novel and powerful architecture for medical image segmentation,^18^ for the identification pancreatic masses. ResNet‐50, a kind of DCNN, was used as a backbone for UNet++ as previously described.^19^, ^20^ Briefly, 3546 images of pancreatic masses marked by an expert in EUS were selected. Afterward, we performed image processing to ensure usability as follows: (1) Divided the image into two parts from the middle mark line, the left panel was the EUS image, and the right panel was the CH‐EUS image, which was included in the original data set; (2) Adjusted the image resolution of the original data set to 512 × 512, then output the map marked by the expert. After that, we divided the original data set into training set and optimization set according to the ratio of 8:2, and performed model training and tuning. TensorFlow was used to build the UNet++ network and completed the training. The whole process is shown in Figure 1. Finally, the prediction confidence of the gland mass area was set to 0.50, the prediction frame pixels were greater than 20, and 886 pancreatic mass images were randomly selected for testing.

#### 2.2.2 Training the model for benign and malignant pancreatic mass identification (Model 2)

**FIGURE 2:**
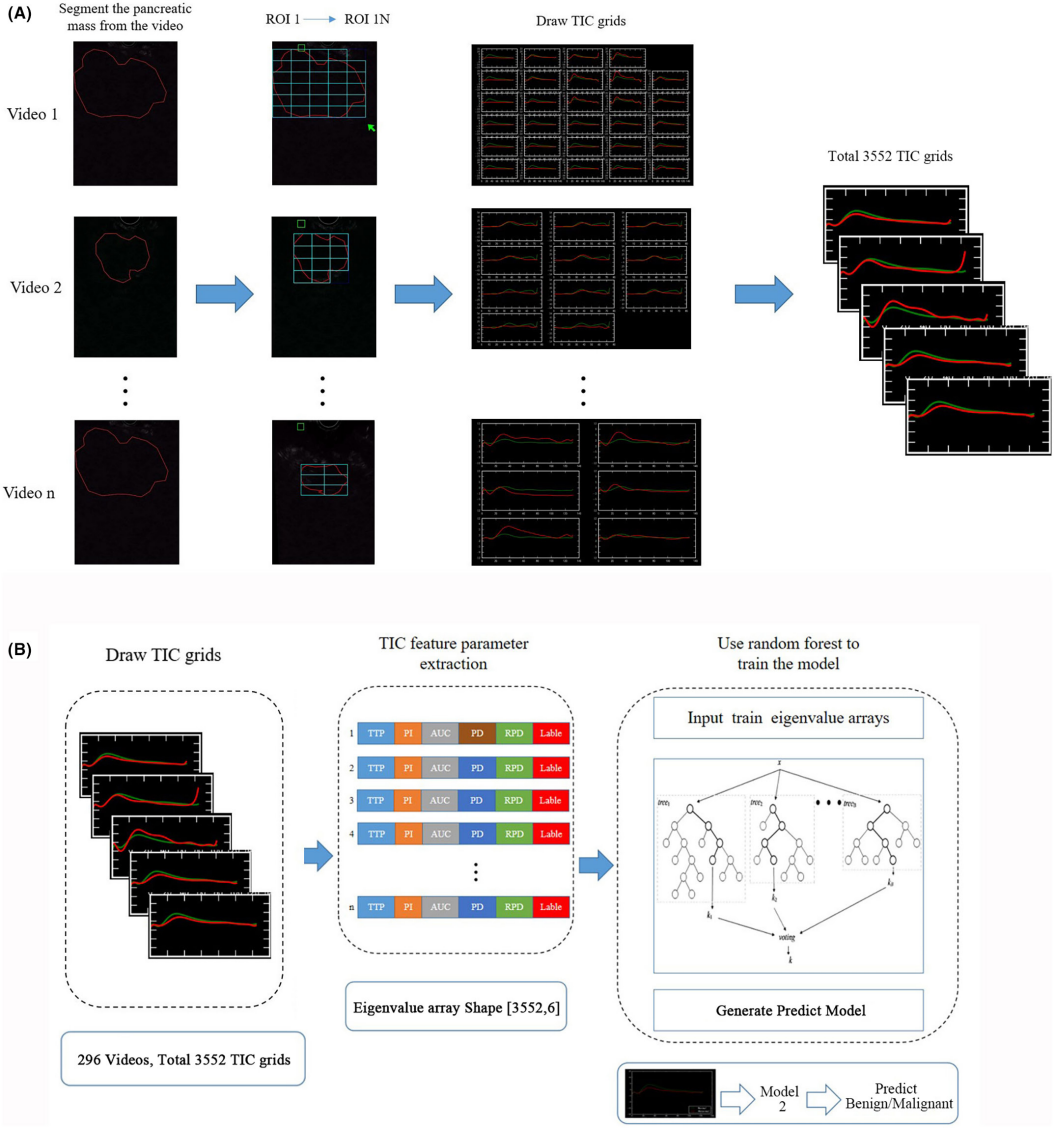
Training the model for benign and malignant pancreatic mass identification (Model 2). (A) Schematic diagram of the process of drawing 3552 TIC grids from 296 CH‐EUS pancreatic mass videos. (B) Schematic diagram of training and testing process of Model 2.

Random Forest, a highly flexible machine learning algorithm has been widely used in the field of medicine,^21^ and was used to train a classification model based on TIC feature analysis to achieve the purpose of differentiating benign and malignant pancreatic masses. A total of 3552 TIC grids from 296 CH‐EUS pancreatic mass videos were used for Model 2 training and testing at the ratio of 8:2. In each video, ROI 1 (the pancreatic mass region) was marked and ROI 2 (the parenchymal region) was selected by an expert of EUS. Then, multiple ROI 1 subregions (ROI 1 N) were segmented and the TIC of each subregion. The detailed process is shown in Figure 2A. And then, the value of five major parameters of TIC: TTP (Peak time of TIC for pancreatic mass), PI (Peak value of TIC for pancreatic mass), AUC (Area under the curve of TIC for pancreatic mass), PD (Peak difference of TIC between pancreatic mass and pancreatic parenchyma), and RPD (The ratio of the peak difference of TIC between pancreatic mass and pancreatic parenchyma) for each TIC grid was extracted and a total of 3552 eigenvalue arrays were obtained. The main process mentioned above is shown in Figure 2B. The prediction confidence of benign and malignant pancreatic masses was set to 0.50, and nine CH‐EUS videos were randomly selected for testing.

### 2.3 Construction and work of CH‐EUS MASTER system

#### 2.3.1 Construction of CH‐EUS MASTER system

After completing the training and testing of Model 1 and Model 2, we constructed the CH‐EUS MASTER System which included the client (real‐time obtainment of images of the main engine of EUS, images processing, model prediction, and results display) and server (network communication, image obtaining from the client, predictions making, and prediction results returning).

#### 2.3.2 Work of CH‐EUS MASTER system

**FIGURE 3:**
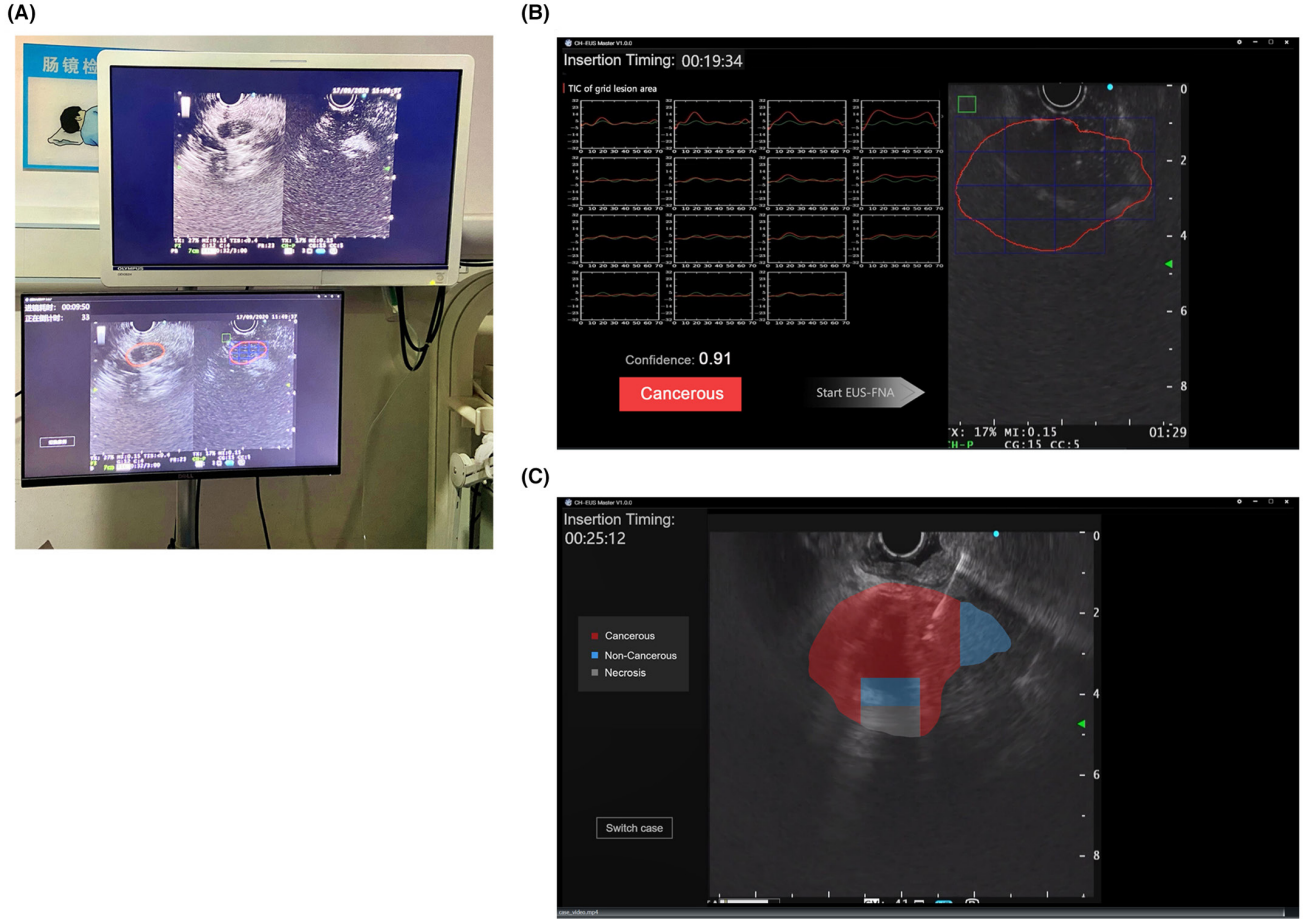
Real‐time use of CH‐EUS MASTER. (A) CH‐EUS MASTER worked with the main engine of EUS, and placed side by side with the original screen, achieving real‐time pancreatic mass capture and segmentation, benign and malignant pancreatic mass identification based on time‐intensity curve analysis and guiding EUS‐FNA. (B) Time–intensity curves (TICs) of regions of interest (ROIs) drawn by CH‐EUS MASTER. In the right panel, the green box indicates the relatively normal parenchymal region (ROI 2), and the red circle indicates the outline of the pancreatic mass (ROI 1), which has been segmented to subregions (ROI 1 N) by blue boxes. Corresponding TICs of ROI 1 N (red lines) and ROI 2 (green lines) are presented in the left panel, which also shows the confidence and the diagnosis of cancerous or noncancerous. (C) Thermogram of the pancreatic mass region according to the time–intensity curves guiding EUS‐FNA. Different color indicates different lesion depending on CH‐EUS MASTER's judgment. Red indicates cancerous, blue indicates noncancerous, and gray indicates necrosis.

After CH‐EUS MASTER was constructed, it worked with the main engine of EUS (Figure 3A). During ordinary ultrasound scanning, CH‐EUS MASTE obtained real‐time images of the main engine of EUS at five frames per second with an average response time of 110 ms for each frame, dynamically identified through Model 1, and marked ROI 1 with a red outline. In the CH‐EUS phase, CH‐EUS MASTE divided ROI 1 into ROI 1 N. At the same time, the doctor selected ROI 2, and drew the TIC grids of ROI 1 N after the CH‐EUS was completed, and then identified the benign and malignant of each area of ROI 1 N by Model 2 and calculated the average confidence of malignancy. When entering the EUS‐FNA phase, CH‐EUS MASTE generated thermogram according to the different properties of each area of ROI 1 N to guide targeting EUS‐FNA.

### 2.4 A single‐center prospective clinical trial

#### 2.4.1 Patients

Consecutive patients with pancreatic masses undergoing CH‐EUS examinations (followed by EUS‐FNA) in the Department of Gastroenterology, The Third Xiangya Hospital of Central South University, China between January and February 2020 were recruited to the study. All patients were followed up for at least 8 months.

##### Inclusion criteria

(1) Adult patients referred for CH‐EUS examinations (EUS‐FNA) after detection of the lesion by transabdominal ultrasound and/or computerized tomography and/or magnetic resonance imaging. (2) Patients agreed to participate in this study and provided signed informed consent.

##### Exclusion criteria

(1) Patients unable to withstand anesthesia or CH‐EUS (EUS‐FNA) examinations; (2) Patients diagnosed with cystic pancreatic tumors or mental disorders as well as those who underwent prior surgical treatment with curative intent or chemoradiotherapy; (3) Adverse events occurring during the trial that affected the observation of efficacy.

##### Withdrawal criteria

Subjects withdrew their informed consent or were lost to follow‐up.

##### Final diagnosis

The diagnosis of malignancy was achieved either by surgical pathology or aspiration cytology/histology with EUS–FNA with clinical follow‐up of at least 8 months.

#### 2.4.2 Procedures and interventions

We prospectively included consecutive patients with pancreatic masses needing EUS‐FNA followed CH‐EUS examination. After traditional EUS, CH‐EUS was performed, two experienced endoscopists watched the screen and determined the nature of the lesion immediately after CH‐EUS, based on the black and white images. Then CH‐EUS MASTER gave the TIC of ROI 1 N and ROI 2, also generated the cancerous or noncancerous judgment of the mass. This study was approved by The Institutional Review Board of The Third Xiangya Hospital of Central South University (protocol number: 2019‐S560).

Patients were then randomly assigned to undergo EUS‐FNA with or without the guidance of CH‐EUS MASTER for the first two passes. In order to ensure patient rights to the best standard‐of‐care and comparability, for the control group, the first two passes were made without CH‐EUS MASTER guidance during FNA, and then two passes were made under guidance from CH‐EUS MASTER; for the CH‐EUS MASTER group, the first two passes were made with CH‐EUS MASTER guidance and then another two manual passes without CH‐EUS MASTER guidance. Only the results of the first two passes were included in the statistical analysis.

These examinations were performed under intravenous anesthesia. All the EUS and follow‐up examinations were performed by experienced endoscopists using ME2 and endoscopes (Olympus Medical Systems Co). The 22‐gauge FNA needles (Cook Medical, Winston Salem, NC; Expect; Boston Scientific, Marlborough, MA, USA) catheter system was used to obtain samples.

##### CH‐EUS

In CH‐EUS, two phases were defined of the pancreas: an early and/or arterial phase (starting from 10 to 30 s) and a late and/or venous phase (from 30 to 90 s). Timing started immediately when the contrast media (sulfur hexafluoride) was injected through left superficial wrist vein slowly. CH mode was selected and the CH‐EUS Doppler image was observed and recorded for 60–90 s.^22^ Two endoscopists gave their independent judgments, then on screen, CH‐EUS MASTER showed the TIC panel, confidence, and diagnosis (cancerous or noncancerous; Figure 3B).

##### EUS‐FNA

Under the guidance of EUS, the needle was visualized in real time and entered the target masses through the wall of the gastrointestinal tract. The endoscopists manually determined the target area (ROI) based on CH‐EUS, punctured four needles at the lesion with a 22‐gauge needle, and each needle was moved back and forth 15–20 times.^23^ For CH‐EUS MASTER‐based guidance of the endoscopist to the puncture, a translucent thermogram was shown on the screen with different colors based on the TIC characteristics of the predicted characteristic of the mass (red indicated malignant, blue indicated benign, and gray indicated necrosis; Figure 3C). After puncturing, each specimen was evaluated by rapid on‐site cytology evaluation (ROSE), histology, and cytology. Specimens were independently evaluated by two histology and pathology experts blinded to the study arms.

#### 2.4.3 Outcomes

The primary outcomes of the study were: (1) to compare the diagnostic accuracy, sensitivity, specificity, positive predictive value (PPV), negative predictive value (NPV), and area under the receiver operator characteristic curve (AUC) of CH‐EUS MASTER and endoscopists in diagnosing benign and malignant pancreatic masses under CH‐EUS; (2) to compare with the diagnostic accuracy, sensitivity, specificity, PPV, NPV, and AUC of the CH‐EUS MASTER group and control group undergoing EUS‐FNA. The secondary outcomes of the EUS‐FNA procedure included first pass of diagnostic sensitivity for pancreatic malignancies, incidence of adverse reactions and complications.

### 2.5 Statistical analysis

Chi‐square (*χ* ^2^) test was used to compare baseline characteristics, the primary outcome, and secondary outcomes. ROC analysis was performed to examine the interaction between sensitivity and specificity. Student's *t* test was used for continuous data like age and numbers of needle passes required for diagnosis which was reported with the mean and standard deviation (SD). *p*‐Value of <0.05 was regarded as statistically significant. All statistical analysis was performed using SPSS for Windows (version 23.0; SPSS Inc.).

## 3 RESULTS

### 3.1 The performance of CH‐EUS MASTER

**FIGURE 4:**
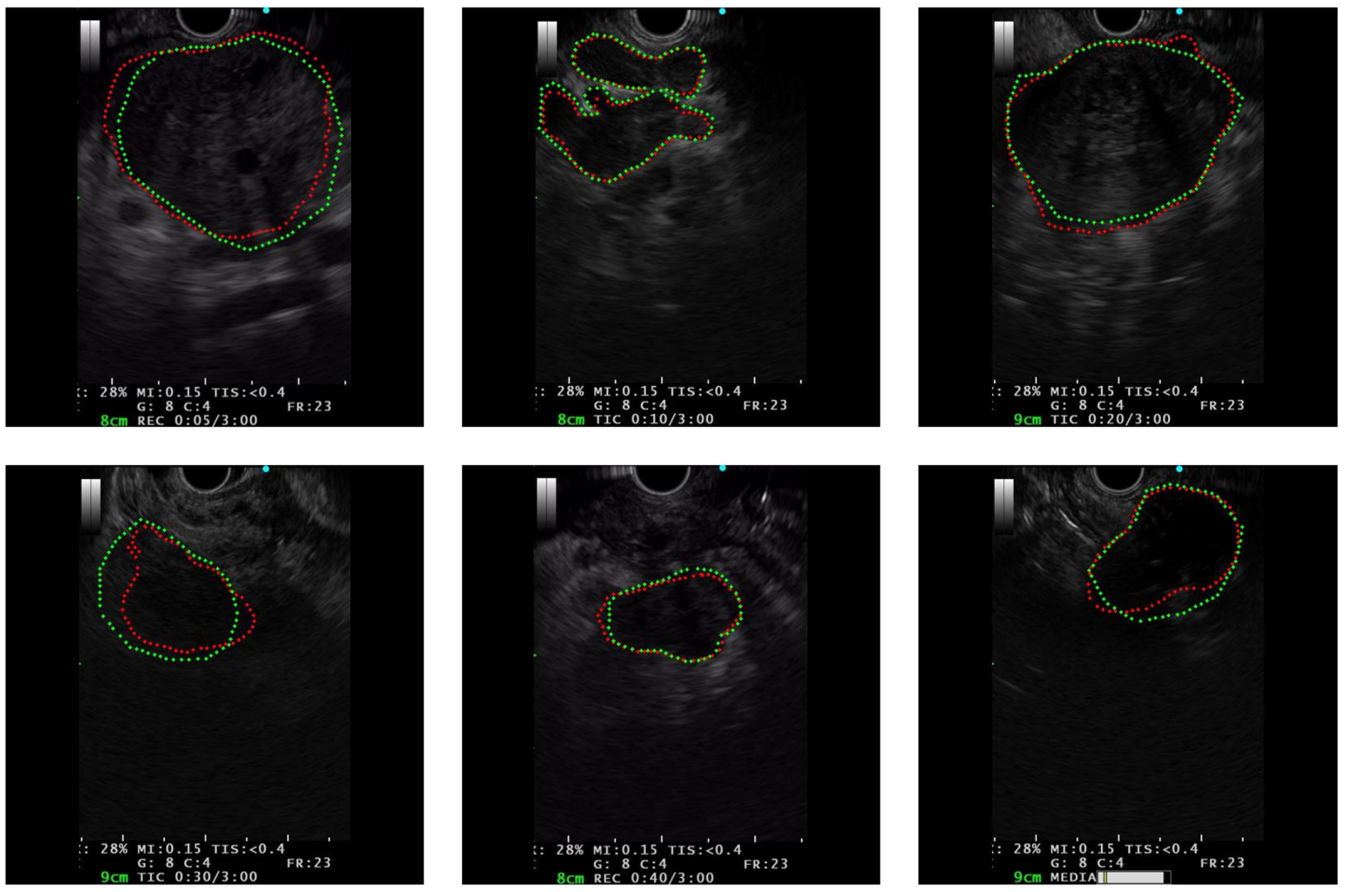
Representative results for Model 1 prediction. In the diagram, the area outlined by the green dashed line is the expert's marked area and the area outlined by the red dashed line is the prediction result area of Model 1.

We constructed a real‐time pancreatic mass capture and segmentation model (Model 1) under CH‐EUS, a benign and malignant identification model (Model 2), and an EUS‐FNA‐targeted auxiliary system. Model 1 successfully identified and segmented the pancreatic mass region of 155 images. Compared with manual labeling by experts, the average IoU (average overlap rate) of Model 1 was 0.708, and the accuracy rate was 87.8% under the overlap rate threshold of 0.50 in CH‐EUS images. Representative results for model prediction are shown in Figure 4.

In the testing set, Model 2 identified malignancies from benign lesions with an accuracy of 88.9%, a sensitivity of 100%, a specificity of 75%, a positive predictive value of 83.3%, and a negative predictive value of 100%.

### 3.2 Patient enrolment and baseline data

**FIGURE 5:**
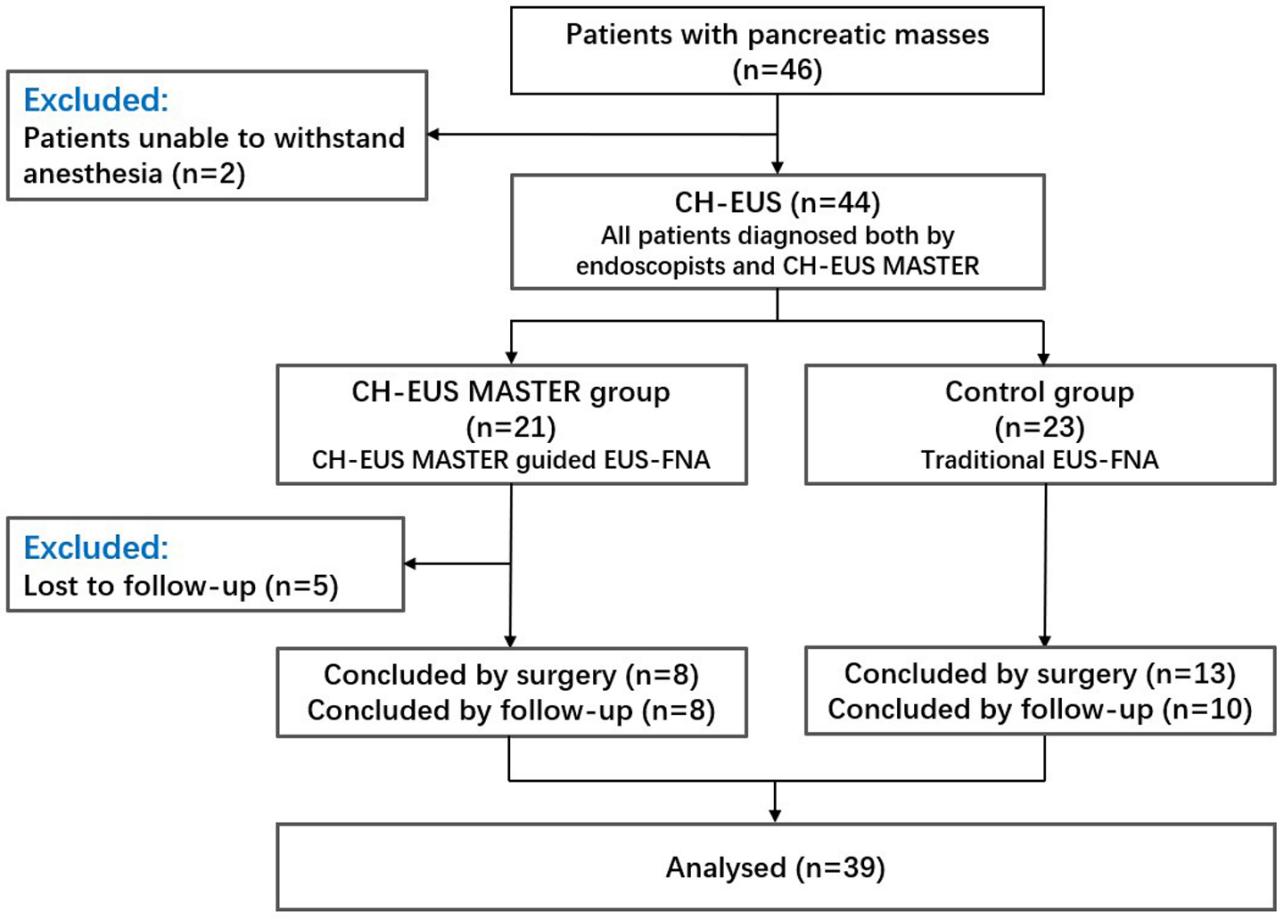
Flowchart of the clinical trial.

Between January and February 2020, 46 patients were recruited, two of them were unable to withstand anesthesia, and five of them were lost to follow‐up. Finally, a total of 39 patients included 26 men and 13 women with a mean age of 58.8 ± 12.2 years (range: 30–81 years) were included. All patients had CH‐EUS examination and were then randomized to undergo either traditional EUS‐FNA (Control group) or CH‐EUS MASTER‐guided EUS‐FNA (CH‐EUS MASTER group). The trial flowchart is illustrated in Figure 5.

The demographic details, laboratory tests, and pancreatic mass baseline characteristics of the included patients are presented in Table 1. There were no statistically significant differences between the groups in these categories. CH‐EUS as well as EUS‐FNA was technically successful in all patients.

**TABLE 1:** The demographic details, laboratory tests, and pancreatic mass baseline characteristics of 39 patients included in the study

Characteristics	CH‐EUS MASTER (*n* = 16)	Control (n = 23)	*p*‐Values
Mean age (SD), years	58.19 ± 12.34	59.30 ± 12.34	0.783
Gender, no. (%)			
Female, no. (%)	5 (31.25)	8 (34.78)	0.818
Male, no. (%)	1 (68.75)	15 (65.22)	
Mass location			
Head/neck, no. (%)	9 (56.25)	14 (60.87)	0.082
Body/tail, no. (%)	2 (12.50)	6 (26.09)	
Ampulla, no. (%)	5 (31.25)	1 (4.35)	
Multiple, no. (%)	0 (0.00)	2 (8.70)	
Maximum diameter (cm)	5.67 ± 8.65	4.17 ± 3.52	0.549
Mass nature			
Malignant, no. (%)	11 (68.75)	15 (65.22)	0.818
Benign, no. (%)	5 (31.25)	8 (34.78)	
Biochemical indicators			
Hemoglobin (g/L)	122.56 ± 21.96	131.24 ± 20.44	0.224
Platelets*10^9^/L	216.13 ± 91.07	190.33 ± 67.20	0.328
WBC*10^12^/L	6.31 ± 2.09	6.45 ± 1.87	0.827
Neutral particle ratio (%)	66.15 ± 9.87	66.77 ± 10.70	0.857
Lymph ratio (%)	23.31 ± 7.49	22.81 ± 10.00	0.870
Total amylase (U/L)	198.94 ± 529.89	82.30 ± 88.24	0.338
Pancreatic amylase (U/L)	172.88 ± 531.91	54.25 ± 85.57	0.332
ALT (U/L)	58.69 ± 84.44	45.67 ± 100.16	0.678
AST (U/L)	57.94 ± 86.30	37.95 ± 43.86	0.364
Albumin (g/L)	37.64 ± 3.47	38.67 ± 7.10	0.600
Globulin (g/L)	25.36 ± 3.42	24.92 ± 4.62	0.755
Direct bilirubin (umol/L)	26.17 ± 61.60	16.50 ± 39.75	0.566
Total bilirubin (umol/L)	40.07 ± 77.98	26.87 ± 46.84	0.526
Creatinine (umol/L)	71.19 ± 29.05	71.90 ± 25.56	0.937
Urea nitrogen (mmol/L)	5.98 ± 2.14	5.78 ± 1.66	0.756
Blood glucose (mmol/L)	6.37 ± 3.12	5.52 ± 0.84	0.248
Triglyceride (mmol/L)	1.47 ± 0.56	1.37 ± 0.63	0.615
Cholesterol (mmol/L)	4.48 ± 1.11	4.64 ± 1.45	0.712
CA199 (U/ml)	181.64 ± 253.72	2231.56 ± 7637.40	0.363
CEA (ng/ml)	2.82 ± 2.40	5.51 ± 3.38	0.084

### 3.3 Comparison between CH‐EUS MASTER and endoscopists in identifying benign and malignant pancreatic masses

Compared with the final diagnosis, there were 24 patients with malignancies and 12 patients with benign lesions correctly identified by CH‐EUS MASTER, while only two cases of PC and one case of pancreatitis were misdiagnosed. While 23 patients with malignancies and 11 patients with benign lesions were correctly identified by endoscopists, three cases of PC and two cases of pancreatitis were misdiagnosed. The accuracy, sensitivity, specificity, PPV, NPV of CH‐EUS MASTER (92.3%, 92.3%, 92.3%, 96.0%, and 85.7%, respectively) were significantly better than that of endoscopists (87.2%, 88.5%, 84.6%, 92.0%, and 78.6%, respectively; *p* < 0.05). The AUC of CH‐EUS MASTER and endoscopists were 0.923 and 0.865, respectively.

### 3.4 Comparison between CH‐EUS MASTER and endoscopists for EUS‐FNA

The procedure success rates were both 100% with no adverse reactions or complications in the two groups. Compared with the final diagnosis, the accuracy, sensitivity, specificity, PPV, and NPV were respectively 93.8%, 90.9%, 100%, 100%, and 83.3% for CH‐EUS MASTER‐guided EUS‐FNA and 91.3%, 85.7%, 100%, 100%, and 81.8% for the control group. Though it seems diagnostic yield was higher in CH‐EUS MASTER group, there was no significant difference between the two groups (*p* > 0.05). The AUC of CH‐EUS MASTER group and control group were 0.955 and 0.933, respectively. The secondary outcomes are presented in Table 2. For subgroup analysis of 22 patients with malignancies, the number of diagnoses of malignancy made in the first pass was significantly greater in the CH‐EUS MASTER group than that in the control group (80.0% vs. 33.3%; *p* = 0.029).

**TABLE 2:** Technical characteristics and outcomes for CH‐EUS MASTER group and control group of EUS‐FNA

Characteristics	CH‐EUS MASTER (*n* = 16)	Control (*n* = 23)	*p* Value
Technical success, no. (%)	16 (100)	23 (100)	
Diagnostic yield, no. (%)	15 (93.8)	20 (87.0)	0.492
No. of diagnoses of malignancy made in first pass (%)^a^	8 (80.0)	4 (33.3)	0.029
Adverse reactions, no. (%)	0 (0)	0 (0)	
Pollution, no. (%)	0 (0)	0 (0)	
Complications, no. (%)	0 (0)	0 (0)	

^a^ Analyzed 22 patients finally diagnosed with malignancies.

## 4 DISCUSSION

Identification and differentiation of pancreatic cancer and mass‐forming chronic pancreatitis is critical and remains a significant problem. Although new methods including EUS, CH‐EUS, and EUS‐FNA have dramatically improved the diagnostic yield of pancreatic masses,^4^, ^6^ the influence of endoscopist's experience and proficiency, and the limitations of the human eye in the analysis of high‐resolution image data are notable. Here, we constructed a novel auxiliary diagnostic AI system, CH‐EUS MASTER, based on the DCNN and Random Forest algorithms, to provide three functions: (1) identifying and tracking pancreatic masses dynamically in real time, (2) differentiating pancreatic cancer from mass‐forming chronic pancreatitis by TIC analysis, and (3) guiding targeted EUS‐FNA.

An emerging body of work demonstrates that deep learning algorithms such as neural network are capable of learning complex features and assisting in diagnosis. In regard to the focus of this study, several different neural networks have been used. For EUS, Das et al. used an artificial neural network (ANN) model to distinguish pancreatic cancer, chronic pancreatitis, and normal pancreatic tissue by digital analysis of EUS images and obtained 93% sensitivity.^24^ Among deep learning techniques, DCNN has been proven to have high performance in medical image analysis and recently has been demonstrated in lung, brain, and breast texture detection.^25^ Here, we established two models (pancreatic mass image segmentation model and pancreatic mass TIC image classification model), retrospectively collected CH‐EUS images and videos for machine learning, and successfully applied the resulting model in clinical practice.

This study is, to the best of our knowledge, the first attempt to develop a real‐time image analysis AI system for the purpose of diagnosing pancreatic masses. This system is especially useful because it can be loaded onto standard CH‐EUS equipment and used to guide EUS‐FNA. The hardest part of Model 1 establish was real‐time image tracking. When Model 1 was integrated into the system, it affected the recognition and segmentation results of pancreatic masses due to image jitter and displacement caused by the patient's breathing and heartbeat. In order to solve this problem, we built a video image stabilization technology based on feature point matching to perform real‐time correction and registration of ultrasound images to prevent real‐time changes from affecting path planning. First, multiple frames of video images and image corners (feature points) were obtained by sequential execution. Second, the optical flow method was used to track the corners. Finally, the affine change matrix representing the motion was obtained according to the changes of the corners of the two images before and after. According to the affine change matrix, the trajectory was calculated and smoothed, then the affine change matrix after smooth motion was obtained, resulting in a stable image. Simply put, this technology ensures the consistency of the results of the previous and subsequent frames when Model 1 performs pancreatic mass recognition and segmentation.

To differentiate pancreatic cancer from mass‐forming chronic pancreatitis, CH‐EUS MASTER performs TIC analysis and gives a confidence value in which values >0.5 are classified as cancerous. Many studies have found that parameters of TIC are useful in the differential diagnosis of focal lesions in pancreatic cancer and chronic pancreatitis. This can be explained by different perfusion patterns at a capillary level in different types of lesions. Due to low vascular density and desmoplasia, pancreatic cancer is typically hypoenhanced compared with the adjacent pancreatic tissue in all phases, while chronic pancreatitis is isoenhanced or hyperenhanced.^26^ Studies using Axius ACQ software to process images from CH‐EUS and obtain TIC, found that time‐dependent parameters (arrival time and time to peak) were significantly longer in pancreatic ductal adenocarcinomas compared to inflammatory masses.^27^ In this study, EUS MASTER greatly improved the accuracy of diagnosing benign and malignant pancreatic masses than endoscopists. CH‐EUS MASTER can calculate TIC parameters effectively and provide a basis for endoscopists to judge benign and malignant pancreatic lesions.

Although EUS‐FNA is currently the gold standard for PC diagnosis, its negative predictive value (NPV) for cancer diagnosis is still insufficient, and the average ratio of the largest series is 70%.^28^ Hou et al.^29^ found that the AUC of CH‐EUS‐guided FNA diagnosis of solid pancreatic lesions was 0.908, with a sensitivity of 81.6%, specificity of 100%, positive predictive value of 100%, negative predictive value of 74.1%, and accuracy of 87.9%. We further used the TIC characteristics to guide FNA. Using CH‐EUS MASTER, a thermogram showing different kinds of lesions based on TIC characteristics is presented on the screen. Endoscopists then punctured the regions of interest (usually presented as a red square/rectangle), and had good outcomes with an accuracy of 93.8%, sensitivity of 90.9%, specificity of 100%, PPV of 100%, and NPV of 83.3%, which was better than previous studies.^4^, ^6^ Importantly, compared to traditional EUS‐FNA, CH‐EUS MASTER‐guided EUS‐FNA can improve the first‐pass diagnostic yield.

This study had several limitations. First, the sample size was small, though we used data augmentation techniques, which guaranteed the training effect of AI with small sample size. Second, patients with other types of focal pancreatic masses (either benign masses like serous cystadenoma or hemangioma as well as malignant tumors such as neuroendocrine tumors, pancreatic metastases, lymphoma, or other rare pancreatic masses) were excluded from the analysis because of the low number of cases. However, multicenter and large sample studies are now underway.

In conclusion, CH‐EUS MASTER can be a promising system for objectively diagnosing malignant and benign pancreatic masses in real time and may guide FNA in real time.

## AUTHOR CONTRIBUTIONS

**Anliu Tang:** Data curation (lead); funding acquisition (lead); methodology (equal); supervision (equal); validation (equal); writing – original draft (lead); writing – review and editing (equal). **Li Tian:** Data curation (equal); funding acquisition (equal); methodology (lead); supervision (equal); validation (equal). **Kui Gao:** Data curation (equal); investigation (equal); validation (equal). **Rui Liu:** Formal analysis (lead); investigation (equal); methodology (equal); validation (equal). **Shan Hu:** Resources (equal); software (lead); supervision (equal); validation (equal); visualization (lead). **Jinzhu Liu:** Methodology (equal); software (equal); validation (equal). **Jiahao Xu:** Data curation (equal); writing – original draft (equal). **Tian Fu:** Data curation (equal); writing – original draft (equal). **Zinan Zhang:** Data curation (equal); formal analysis (equal). **Wujun Wang:** Software (equal); validation (equal); visualization (equal). **Long Zeng:** Software (equal). **Weiming Qu:** Resources (equal). **Yong Dai:** Resources (equal). **Ruirui Hou:** Data curation (equal). **Shoujiang Tang:** Writing – review and editing (equal). **Xiaoyan Wang:** Project administration (lead); supervision (lead); writing – review and editing (lead).

## DISCLOSURE

None declared.

## DISCLAIMER

The funding sources did not have any influence on study design, data collection, analysis and interpretation of the data, writing the report, or the decision to submit for publication.

## PATIENT CONSENT FOR PUBLICATION

Obtained.

## FUNDING INFORMATION

State key clinical specialty of China, Hunan Provincial Science & Technology Department of China (2020SK2013), and Hunan Provincial Natural Science Foundation of China (2021JJ40946).

## ETHICAL APPROVAL

This study was approved by The Institutional Review Board of The Third Xiangya Hospital of Central South University (protocol number: 2019‐S560).

## Data Availability

Data sharing is not applicable to this article as no new data were created or analyzed in this study.
